# Enhancing bowel sound recognition with self-attention and self-supervised pre-training

**DOI:** 10.1371/journal.pone.0311503

**Published:** 2024-12-31

**Authors:** Yansuo Yu, Mingwu Zhang, Zhennian Xie, Qiang Liu

**Affiliations:** 1 Academy of Artificial Intelligence, Beijing Institute of Petrochemical Technology, Beijing, China; 2 Xiyuan Hospital, Chinese Academy of Traditional Chinese Medicine, Beijing, China; Jordan University of Science and Technology Faculty of Computer and Information Technology, JORDAN

## Abstract

Bowel sounds, a reflection of the gastrointestinal tract’s peristalsis, are essential for diagnosing and monitoring gastrointestinal conditions. However, the absence of an effective, non-invasive method for assessing digestion through auscultation has resulted in a reliance on time-consuming and laborious manual analysis by clinicians. This study introduces an innovative deep learning-based method designed to automate and enhance the recognition of bowel sounds. Our approach integrates the Branchformer architecture, which leverages the power of self-attention and convolutional gating for robust feature extraction, with a self-supervised pre-training strategy. Specifically, the Branchformer model employs parallel processing of self-attention and convolutional gated Multi-layer Perceptron branches to capture both global and local dependencies in audio signals, thereby enabling effective characterization of complex bowel sound patterns. Furthermore, a self-supervised pre-training strategy is employed, leveraging a large corpus of unlabeled audio data to learn general sound wave representations, followed by fine-tuning on a limited set of bowel sound data to optimize the model’s recognition performance for specific tasks. Experimental results on public bowel sound datasets demonstrate the superior recognition performance of the proposed method compared to existing baseline models, particularly under data-limited conditions, thereby confirming the effectiveness of the self-supervised pre-training strategy. This work provides an efficient and automated solution for clinical bowel sound monitoring, facilitating early diagnosis and treatment of gastrointestinal disorders.

## Introduction

Bowel sounds (BS), the direct reflection of gastrointestinal motility, are sporadic noises originating from the gas and fluid movement within the intestines. These intermittent sounds hold substantial significance in assessing the health status of the human gastrointestinal tract [1, 2]. The genesis mechanism of these bowel sounds is intrinsically tied to the dynamic interplay of gas and fluid within the gut, thereby embodying its diverse physiological and pathological states. In the realm of clinical practice, the surveillance of these bowel sounds plays a critical role in diagnosing and monitoring numerous gastrointestinal diseases, such as intestinal obstruction, inflammatory bowel disease, and dyspepsia. However, the study and clinical application of bowel sounds are limited due to their seemingly random nature, wide dynamic range, and potential dietary influences. Conventionally, the gathering and scrutiny of bowel sounds depend on subjective auscultation by medical professionals [3, 4]. This approach, while instinctual and pervasive, is vulnerable to intervention from environmental noise and constrained by the doctor’s personal experience, and the brevity of data collection period. Consequently, it might compromise the accuracy and reliability of the outcomes.

The research background of bowel sound recognition technology dates back to early acoustic analysis methods [5, 6], which primarily relied on traditional signal processing techniques such as Fourier transform to analyze bowel sound signals. Over time, researchers began to explore machine learning algorithms [7], such as Support Vector Machine (SVM) [8] and Naive Bayesian [9], to enhance the automation level of recognition. For instance, Yin Y et al. [8] proposed a bowel sound recognition method based on SVM, which employs Legendre polynomial fitting on the logarithmic amplitude spectrum to effectively filter out non-essential information. This method ingeniously applies Principal Component Analysis (PCA) to dimensionally reduce acoustic features within a specific frequency range, and further optimizes the SVM model with the least squares method and radial basis function kernel, significantly enhancing recognition accuracy. Ulusar [9] utilized a Naive Bayes algorithm for bowel sound pattern classification, coupled with minimum statistics and spectral subtraction techniques for noise reduction, achieving satisfactory results in actual audio recording tests. However, these traditional methods [10, 11] depend on specialized knowledge for feature extraction and struggle to adapt to the complex variations in bowel sound signals.

The advent of Deep Neural Networks (DNN) has marked a significant paradigm shift [12–15] in the domain of bowel sound recognition. Convolutional Neural Networks (CNN), in particular, have harnessed the power of spatial feature extraction in audio signals, capitalizing on their local receptive fields and weight sharing efficiencies [16–18]. Zhao et al. [17] explored a variety of CNN architectures for BS detection within 5-second audio segments, albeit without sufficient detail for method replication. A subsequent study [19] employed a CNN tailored to 1-second segments, achieving promising results with 1-minute audio clips from 28 participants under conditions of maximum Signal-to-Noise Ratio (SNR). Recurrent Neural Networks (RNN), especially Long Short-Term Memory (LSTM) networks [20], have garnered attention for their proficiency in capturing temporal dynamics within BS signals, a testament to their prowess in time-series data analysis. Liu et al. [21] exemplified this by training an LSTM on Mel-Frequency Cepstral Coefficients (MFCC), using non-overlapping windows to identify BS sections exceeding 100 milliseconds. However, they noted a decline in performance amidst varying environmental noise. Moreover, the fusion of CNN and RNN in hybrid models [22, 23] has propelled recognition performance to new heights. These models offer an innovative pathway for the automatic classification and analysis of bowel sounds, adeptly integrating both spatial and temporal features to enhance diagnostic accuracy and reliability.

Research on bowel sound recognition technology has gained increasing attention in recent years, yet it continues to faces numerous challenges. On one hand, current models are deficient in automatic feature extraction and handling of long-term dependencies. Although models such as CNNs [24] and LSTMs [21] have achieved certain successes in specific tasks, they are limited in capturing the subtle changes and long-term patterns of bowel sound signals. Moreover, the training process of these models [25, 26] often requires extensive manual intervention and feature engineering, which not only increases the complexity of research but also limits the models’ generalization capabilities. On the other hand, there is a relative scarcity of high-quality bowel sound data. The collection of bowel sound data is complex and costly, and the data quality is influenced by various factors, such as the precision of sensors, changes in the subject’s body position, and dietary habits. These factors lead to inconsistent data quality, adding to the difficulty of analysis and recognition.

Recently, the attention mechanism [27, 28] has emerged as a pivotal force driving technological advancements within the field of deep learning. It plays a significant role across various sub-domains such as computer vision [29, 30], speech recognition [31], and natural language processing [32–34]. The attention mechanism [27], a technique within artificial neural networks that simulates human cognitive attention [35], adjusts the network’s focus on different parts of the input data, thereby endowing the neural network with the capability to concentrate on key features within the data. This mechanism amplifies the weight of the more important aspects of the data while diminishing the influence of others, allowing the network’s focus to be dynamically allocated according to the context’s requirements. Concurrently, the rise of self-supervised pre-training methods [32, 33, 36] has effectively addressed the issue of insufficient labeled data. These methods leverage a vast amount of unlabeled data to extract features with broad applicability, significantly enhancing the model’s generalization capabilities. Notably, pre-trained models like Bidirectional Encoder Representations from Transformers (BERT) [33] have mitigated reliance on extensive manually annotated datasets, bolstering the model’s adaptability to specific tasks after fine-tuning, thus sparking a transformation in the field of natural language processing.

Building upon these technological breakthroughs, this study introduces an innovative method for bowel sound recognition. The method integrates advanced attention mechanisms with self-supervised pre-training strategies, aiming to surmount the challenges of bowel sound recognition under data-limited conditions. Firstly, a bowel sound recognition method based on Branchformer [37, 38], which leverages the parallel processing capabilities of its self-attention and Multi-layer Perceptron with convolutional gating (cgMLP) modules to effectively capture both global and local features of audio signals, thus enabling a deeper understanding of bowel sounds. Secondly, a self-supervised pre-training strategy [33, 34] is introduced. This strategy involves pre-training on a large amount of unlabeled audio data to learn a universal representation of sound waves [39, 40], followed by fine-tuning on a limited set of high-quality bowel sound datasets to optimize the model’s recognition performance for specific tasks. This integrated approach is expected to improve the accuracy and efficiency of bowel sound analysis, offering new insights and tools for medical diagnostics.

The research contributions of this paper are primarily reflected in the following aspects:

- A bowel sound recognition method based on Branchformer is proposed, which effectively captures both global and local features of audio signals through its parallel branch architecture, enhancing the model’s understanding of bowel sound signals.
- A self-supervised pre-training strategy is introduced, which addresses the issue of insufficient high-quality bowel sound data by pre-training on unlabeled audio data, thereby improving the model’s generalization capabilities.
- Fine-tuning on a limited set of high-quality bowel sound datasets optimizes the model’s performance for specific recognition tasks, enabling the training of high-performance bowel sound recognition models even when labeled data is scarce.
- The effectiveness of the proposed methods is validated through experiments, demonstrating the significant advantages of the Branchformer model and self-supervised pre-training strategy over existing technologies in the task of bowel sound recognition.

The structure of this paper is as follows: The second section will provide a detailed introduction to the proposed bowel sound recognition method based on Branchformer and the specific implementation of the self-supervised pre-training strategy; The third section will present the experimental results and compare them with existing technologies; Finally, the fourth section will summarize the paper and discuss future research directions.

## Materials and methods

**Fig 1:**
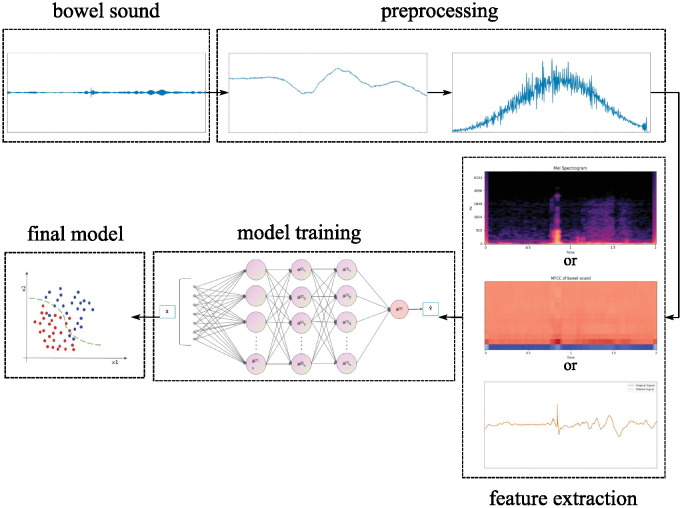
Training flowchart of bowel sound recognition.

The comprehensive methodology for training the bowel sound recognition model, as shown in Fig 1, was conducted over an eighteen-month period and includes data collection, preprocessing, and model training stages. The model training procedure is structured into sequential steps: acquisition of raw data, preprocessing, acoustic feature extraction, and model development. During the initial analysis phase, two categories of samples are identified: bowel sound events and non-bowel sound events, both annotated by medical professionals. The preprocessing phase is meticulously executed through four critical steps—segmentation, normalization, pre-emphasis, and window framing—to enhance signal fidelity and mitigate interference with the deep learning model. Following preprocessing, the focus shifts to the extraction of acoustic features from each bowel sound signal frame, a pivotal phase in the development of the recognition model. With the requisite features secured, the model training phase commences, utilizing a partitioned dataset comprising training, testing, and validation subsets to ensure robust training, model validation, and performance assessment.

The selection of parameters, including sampling rate, frame length, frame shift, and window function, plays a crucial role in effectively capturing and representing the intricate characteristics of bowel sound signals. The sampling rate of 44.1kHz was chosen to preserve the high-fidelity information present in the original audio recordings, ensuring accurate representation of the subtle nuances within the bowel sound signals. Additionally, evaluating the model’s performance across varying sampling rates (8kHz and 22.05kHz) allowed for assessing its robustness and adaptability to diverse audio sources. The frame length of 25ms strikes an optimal balance between capturing sufficient signal information and maintaining computational efficiency, aligning with established practices in audio signal processing. The frame shift, or hop length, of 10ms facilitates a reasonable overlap between consecutive frames, enabling the model to effectively capture temporal dependencies while mitigating excessive redundancy. Furthermore, the Hamming or Hanning window function was employed to reduce spectral leakage and minimize discontinuities at frame boundaries, thereby minimizing artifacts in the frequency domain representation, a common consideration in audio processing applications.

### Dataset preparation

The public bowel sound dataset used in this study comes from [23], covering a total of 19 participants’ bowel sound records and compiling 1,605 audio files, each with a duration of 2 seconds. These audio files were collected using a device equipped with a dedicated contact microphone and were saved in mono-channel WAV format with a sampling rate of 44.1kHz and a sampling depth of 24 bits. In addition, each audio file is accompanied by a corresponding CSV format label file containing information on bowel sound event locations, highest and lowest frequencies. We have processed the audio data with the librosa [41] library in Python and aligned it synchronously with the labeled data, resulting in a total of 6,378 data samples. Among these samples, 3,699 were marked as bowel sound fragments, and 2,679 were marked as non-bowel sound fragments. This classification helps to increase the balance between categories in the dataset thus avoiding any form of bias or skew. In addition, to maintain training consistency and reduce potential impacts due to volume differences, the amplitudes of all samples were normalized to a range of [-1,1]. As such, the adaptability of the model has been enhanced, allowing it to better handle data under different recording conditions, thereby enhancing its generalization ability.

### Framework of the Branchformer model

Branchformer is an innovative encoder architecture primarily designed for speech recognition and understanding tasks [37, 38]. It captures both local and global context information through parallel multi-layer perceptrons (MLPs) and attention mechanisms. The main advantages of this architecture lie in its flexibility, interpretability, customizability, and efficient computational performance. Compared to traditional RNNs, it can better parallelize data processing and more effectively capture long-distance dependencies. Unlike CNNs, it extends its focus beyond local dependencies to encompass global context, facilitated by the self-attention mechanism, which is crucial for processing continuous speech data. Additionally, Branchformer surpasses Transformers [27] by enhancing local feature extraction via the Multi-layer Perceptron with convolutional gating (cgMLP) module and by incorporating interchangeable self-attention variants to enhance flexibility and efficiency.

**Fig 2:**
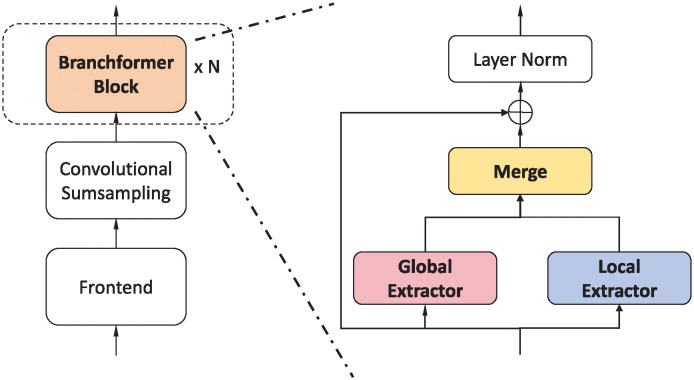
Overall architecture of the Branchformer encoder. A stack of identical Branchformer blocks are used to capture local and global dependencies.

The overall architecture of the Branchformer encoder, as illustrated in Fig 2, begins with a frontend module designed to process the raw audio sequence and extract low-level acoustic features. Subsequently, a convolutional subsampling module is engaged to temporally downsample the feature sequence. The encoder is composed of N sequentially stacked, identical Branchformer blocks, each adept at capturing both global and local feature dynamics. Within each Branchformer block, three key components function in concert: a global extractor branch, a local extractor branch, and a merging module that integrates their outputs. The global feature extractor operates via a self-attention mechanism tailored to identify long-range dependencies, whereas the local feature extractor operates through a cgMLP to capture nuanced local interactions. This dual-branch structure allows the model to dynamically balance the importance of local and global contexts across layers, enhancing overall performance.

#### Global extractor branch

**Fig 3:**
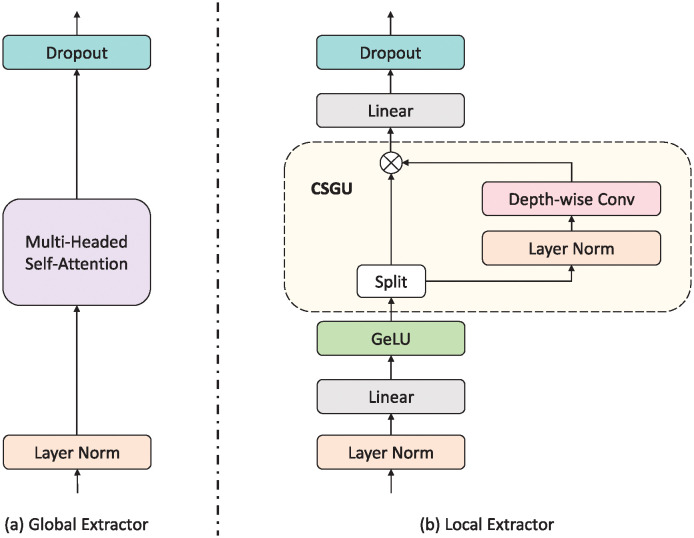
Global extractor and local extractor in Branchformer. Global extractor employs attention to capture global context, while local extractor utilizes the MLP with convolutional gating to extract local context.

The global extractor branch within the Branchformer architecture, as shown in Fig 3(a), is designed to capture the global context of the input sequence through a self-attention mechanism. This branch operates on the premise of the Transformer’s pre-norm [42] setup, where a layer norm (LN) [43], multi-head self-attention (MHSA) [27], and dropout [44] are applied sequentially. The input to this branch is a feature sequence $X\in R^{T\times d}$, which is transformed into query, key, and value matrices $Q,K,V\in R^{T\times d}$. The self-attention mechanism calculates the scaled dot product between queries and keys, resulting in a weighted combination of values that represent the global context $Y_{G}\in R^{T\times d}$. This process is encapsulated in the following equations:

$$\begin{array}{c} Y_{G}=\text{Dropout}\left(\text{MHSA}\left(\text{LN}\left(X\right)\right)\right). \end{array}$$ (1)

#### Local extractor branch

The local extractor branch, as illustrated in Fig 3(b), focuses on the local context within the sequence, achieved through a module known as cgMLP [45]. This module uses depth-wise convolution and linear gating to process the input features and capture local dependencies. The cgMLP consists of channel projections, a Gaussian Error Linear Unit (GELU) activation [46] function, a convolutional spatial gating unit (CSGU) [45], and additional channel projections. The local context $Y_{L}\in R^{T\times d}$ is extracted through a series of operations as follows:

$$\begin{array}{c} Z=\text{GELU}(\text{LN}(X)U), \end{array}$$ (2)

$$\begin{array}{c} {}[A\;B]=Z, \end{array}$$ (3)

$$\begin{array}{c} \tilde{Z}=\text{CSGU}\left(Z\right)=A⊙\text{DwConv}\left(\text{LN}\left(B\right)\right), \end{array}$$ (4)

$$\begin{array}{c} Y_{L}=\text{Dropout}\left(\tilde{Z}V\right). \end{array}$$ (5)

where $Z\in R^{T\times d_{\text{inter}}}$, $A,B,\tilde{Z}\in R^{T\times d_{\text{inter}}/2}$ are intermediate hidden features, and $U\in R^{d\times d_{\text{inter}}}$, $V\in R^{d_{\text{inter}}/2\times d}$ denote the trainable weights of two linear projections.

#### Merging branches

The Branchformer encoder merges the outputs from both the global and local branches using either concatenation or weighted average methods. The concatenation method is the default, where the outputs *Y*_*G*_ and *Y*_*L*_ are combined and then projected back to the original dimension *d* using a linear projection $W\in R^{2d\times d}$. This is represented by the following equation:

$$\begin{array}{c} Y_{\text{Merge}}=\text{Concat}\left(Y_{G},Y_{L}\right)W. \end{array}$$ (6)

Alternatively, a weighted average method can be employed, where the model dynamically generates weights for each branch, providing a more interpretable representation of how global and local dependencies are integrated. This method allows for a flexible and customizable approach to merging the branches’ outputs.

#### Complexity analysis

The complexity of the Branchformer architecture is analyzed with respect to sequence length *T* and feature dimension *d*. The attention-based branch, which may incorporate self-attention or the Fastformer mechanism, has a complexity of *O*(*T*^2^*d*) for standard self-attention and *O*(*Td*) for Fastformer. The cgMLP branch operates with linear complexity *O*(*Td*), making it efficient for processing sequences of varying lengths. Moreover, the Branchformer model employs strategies aimed at reducing computational complexity, such as the incorporation of branch dropout during the training phase. This approach expedites inference processes without significantly compromising the model’s accuracy.

### Strategy of self-supervised pre-training

**Fig 4:**
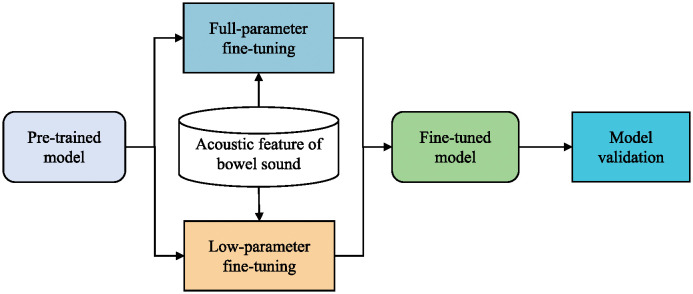
Training process diagram of the bowel sound recognition model based on self-supervised pre-training.

Given the limited availability of high-quality bowel sound data, this study adopts a self-supervised pre-training strategy to enhance the model’s understanding of bowel sound signals. The training process of the bowel sound recognition model based on self-supervised pre-training is depicted in Fig 4.

Initially, in the pre-training phase, the model is trained on unlabeled audio data. By designing various self-supervised tasks, such as predicting future frames or reconstructing masked segments, the model learns a universal representation of sound. Two pre-training models were selected for this study: HuBERT [39] and wav2vec 2.0 [40]. Both are self-supervised speech pre-training models capable of learning useful representations from raw audio. These pre-trained models are then fine-tuned on a limited set of high-quality bowel sound datasets to adapt to specific recognition tasks. This strategy not only improves the model’s ability to recognize bowel sound signals but also alleviates the reliance on a large amount of labeled data. Building upon the pre-trained models, this paper further implements two fine-tuning strategies: full-parameter fine-tuning and low-parameter fine-tuning. Full-parameter fine-tuning involves optimizing all parameters of the pre-trained model to better adapt to the specific bowel sound recognition task. Although this method can achieve higher recognition accuracy, it comes with higher computational costs. To address this issue, this paper also explores a low-parameter fine-tuning method, which optimizes only a portion of the model’s parameters to lighten the model while maintaining high recognition performance.

**Fig 5:**
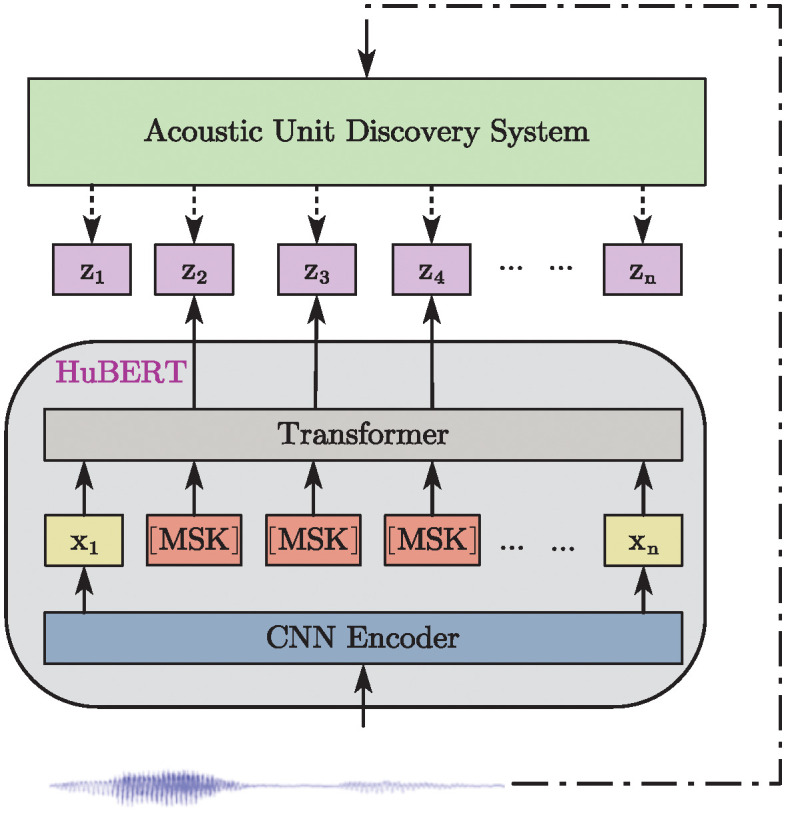
Illustration of HuBERT, which predicts hidden cluster assignments of the masked frames.

**Fig 6:**
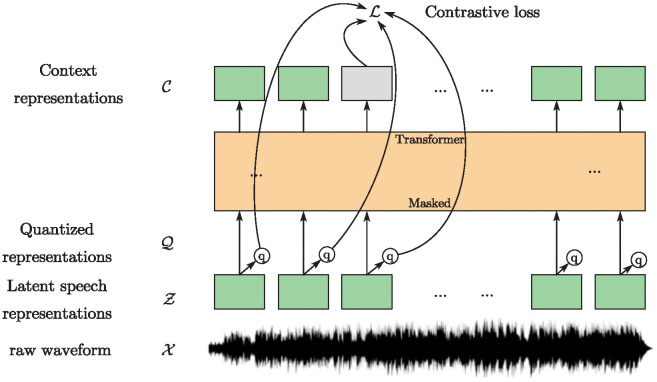
Illustration of wav2vec 2.0, which jointly learns contextualized acoustic representations and an inventory of discretized acoustic units.

- HuBERT [39], as shown in Fig 5, is a novel self-supervised speech representation learning model that employs a clustering-based approach to generate pseudo-labels from raw audio and utilizes these pseudo-labels for self-supervised training. This method enables HuBERT to effectively learn complex patterns and structures within audio signals, thereby excelling in tasks such as speech recognition. By undergoing pre-training and fine-tuning, HuBERT adapts to specific speech processing tasks, thereby enhancing recognition accuracy and efficiency.
- wav2vec 2.0 [40], as shown in Fig 6, is an advanced self-supervised speech pre-training model that effectively captures important features of speech by training on a large amount of unlabeled audio data. The model’s training process consists of two main stages: pre-training and fine-tuning. During the pre-training phase, the model learns to encode raw audio waveforms and acquires useful representations by addressing masking tasks within the audio. The fine-tuning phase then employs a small amount of labeled data to adjust the model for specific speech recognition tasks.

## Results

To evaluate the performance of the bowel sound recognition model, this paper employs the calculation of accuracy as the experimental evaluation metric. The formula for calculating accuracy is as follows:

$$\begin{array}{c} ACC=\frac{TP+TN}{P+N} \end{array}$$ (7)

Where TP represents the number of samples that belong to the bowel sound category and are correctly identified as such by the recognition model, TN represents the number of samples that belong to the non-bowel sound category and are correctly identified as such by the recognition model, P represents the total number of samples that belong to the bowel sound category, and N represents the total number of samples that belong to the non-bowel sound category.

### Performance comparison of various prediction models

In this study, a series of comprehensive experimental schemes were devised to deeply evaluate the performance of various deep learning models in the task of bowel sound recognition. The recognition performance of each model was systematically assessed under various conditions, including processing multiple acoustic features, different sampling rates, frame lengths, frame shifts, window functions, and feature lengths. The acoustic features utilized in the experiments included Mel-frequency cepstral coefficients (MFCC), Linear Predictive Coding (LPC), and Mel-Spectrum. In this series of experiments, we selected four representative deep learning models, namely Convolutional Neural Networks (CNN), Long Short-Term Memory networks (LSTM), a combined model of CNN and LSTM (CNN+LSTM), and ResNet34 [47].

**Fig 7:**
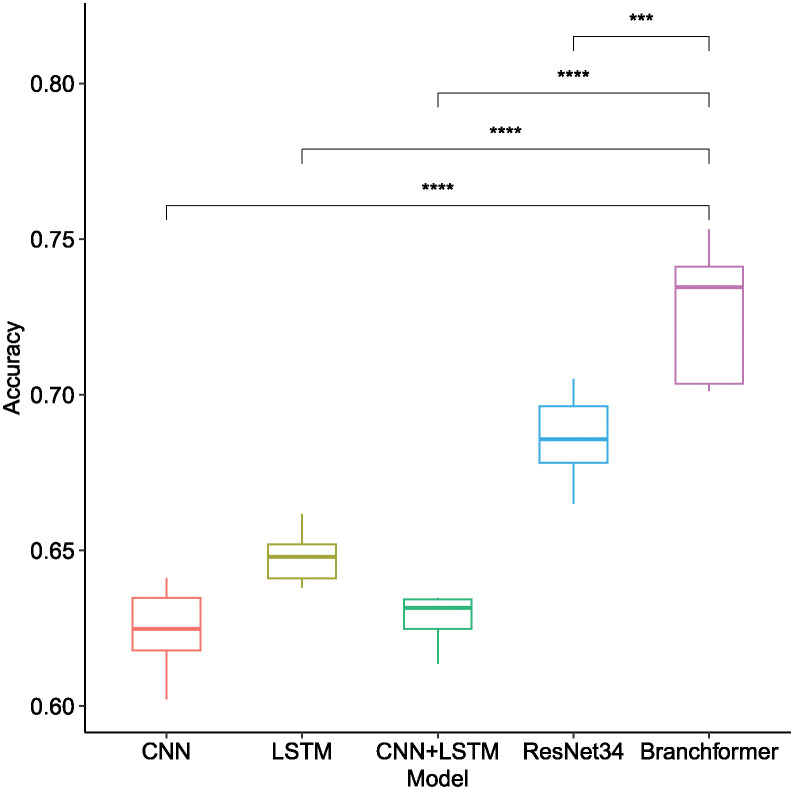
Box plot of recognition accuracy among different model architectures when utilizing different acoustic features of bowel sound signals across different sampling rates. Horizontal bars depict Mann–Whitney U tests for significance of differences in accuracy between Branchformer and other models. **p-value ≤ 0.01, ***p-value ≤ 0.001, ****p-value ≤ 0.0001.

As illustrated in Table 1 and Fig 7, the Branchformer model demonstrated consistent outperformance relative to other models across a variety of sampling rates and feature types, thereby showcasing its superiority. Utilizing Mel-spectrogram features, the Branchformer model secured remarkable accuracy scores, varying from 0.7012 to 0.7498, corresponding to sampling rates of 8 kHz, 22.05 kHz, and 44.1 kHz, respectively. This performance advantage was statistically significant, as indicated by the distinct separation of boxplot whiskers and the associated low p-values. While the ResNet34 model delivered commendable results within the Mel-spectrogram feature range of 0.6807 to 0.6973, it was outperformed by the Branchformer. The trend observed was that increased sampling rates generally enhanced accuracy for the majority of models and features, presumably because of the greater detail captured in the signal. The Branchformer’s consistent excellence across all sampling rates confirms its robustness and proficiency in managing the acoustic characteristics of bowel sounds.

**Table 1:** Comparative analysis of recognition accuracy for different model architectures using different acoustic features of bowel sound signals across different sampling rates. Note that *sr* indicates the sampling rate.

Model	Acoustic Feature	*ACC*_*sr*=8*kHz*_	*ACC*_*sr*=22.05*kHz*_	*ACC*_*sr*=44.1*kHz*_
CNN	MFCC	0.6179	0.6348	0.6296
	LPC	0.6359	0.6248	0.6412
	Mel Spectrum	0.6022	0.6151	0.6235
LSTM	MFCC	0.6513	0.6519	0.6479
	LPC	0.6381	0.6411	0.6409
	Mel Spectrum	0.6433	0.6617	0.6891
CNN+LSTM	MFCC	0.6137	0.6534	0.6347
	LPC	0.6312	0.6248	0.6169
	Mel Spectrum	0.6315	0.6322	0.6343
ResNet34	MFCC	0.6973	0.7051	0.6963
	LPC	0.6782	0.6754	0.6651
	Mel Spectrum	0.6807	0.6857	0.6917
Branchformer	MFCC	**0.7356**	**0.7532**	0.7412
	LPC	0.7028	0.7036	0.7146
	Mel Spectrum	0.7012	0.7346	**0.7498**

**Fig 8:**
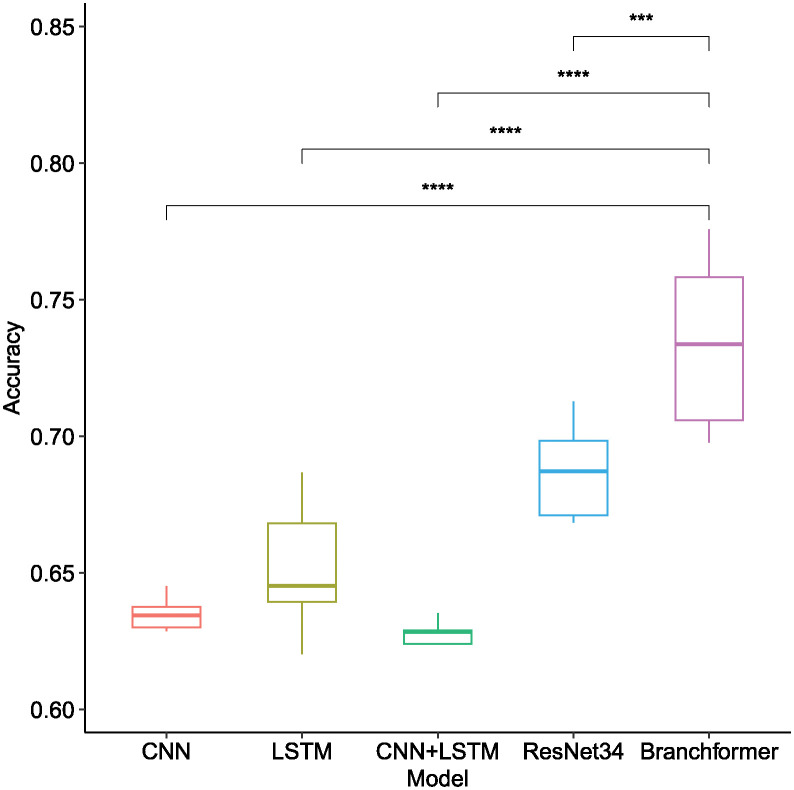
Box plot of recognition accuracy among different model architectures when utilizing different acoustic features of bowel sound signals across different frame lengths. Horizontal bars depict Mann–Whitney U tests for significance of differences in accuracy between Branchformer and other models. **p-value ≤ 0.01, ***p-value ≤ 0.001, ****p-value ≤ 0.0001.

As depicted in Table 2 and Fig 8, the Branchformer model demonstrated consistent outperformance compared to other models across various feature types and frame lengths, with notably higher accuracy. Specifically, utilizing Mel-spectrogram features, the Branchformer model secured accuracy scores between 0.7584 and 0.7759 across all frame lengths, a statistically significant advantage as indicated by the distinct separation of boxplot whiskers and the corresponding low p-values. While the ResNet34 model provided commendable results within the Mel-spectrogram feature range of 0.6852 to 0.6984, it was outperformed by the Branchformer. The Mel-spectrogram features generally outperformed MFCC and LPC in capturing the nuances of bowel sounds, enhancing accuracy across the majority of models. The impact of frame length on model accuracy was pronounced, yet the Branchformer maintained remarkable stability and precision across all frame lengths, particularly in conjunction with Mel-spectrogram features.

**Table 2:** Comparative analysis of recognition accuracy for different model architectures using different acoustic features of bowel sound signals across different frame lengths. Note that *fl* indicates the frame length.

Model	Acoustic Feature	*ACC*_*fl*=1024_	*ACC*_*fl*=1323_	*ACC*_*fl*=1764_
CNN	MFCC	0.6345	0.6396	0.6301
	LPC	0.6288	0.6337	0.6356
	Mel Spectrum	0.6180	0.6452	0.6377
LSTM	MFCC	0.6431	0.6682	0.6371
	LPC	0.6203	0.6454	0.6395
	Mel Spectrum	0.6638	0.6742	0.6868
CNN+LSTM	MFCC	0.6123	0.6284	0.6245
	LPC	0.6353	0.6291	0.6038
	Mel Spectrum	0.6241	0.6304	0.6289
ResNet34	MFCC	0.6873	0.7128	0.7010
	LPC	0.6685	0.6711	0.6703
	Mel Spectrum	0.6984	0.6945	0.6852
Branchformer	MFCC	0.7204	0.7337	0.7559
	LPC	0.7059	0.6978	0.7034
	Mel Spectrum	**0.7584**	**0.7759**	**0.7628**

**Fig 9:**
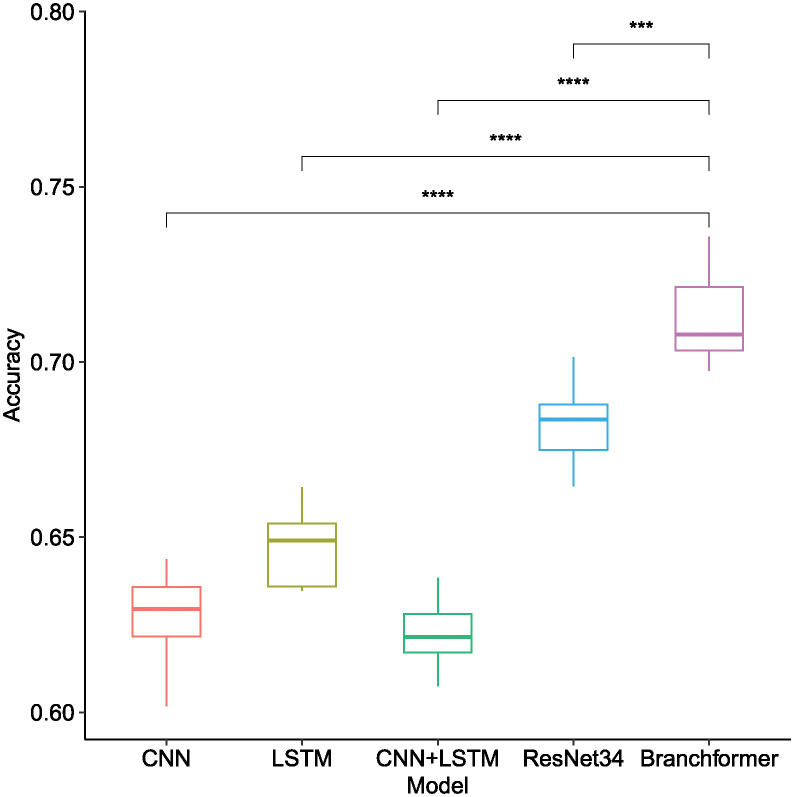
Box plot of recognition accuracy among different model architectures when utilizing different acoustic features of bowel sound signals across different frame shifts. Horizontal bars depict Mann–Whitney U tests for significance of differences in accuracy between Branchformer and other models. **p-value ≤ 0.01, ***p-value ≤ 0.001, ****p-value ≤ 0.0001.

As illustrated in Table 3 and Fig 9, the Branchformer model demonstrated consistent outperformance relative to other methods across various feature types and frame shift sizes, thereby showcasing its superior accuracy. Utilizing Mel-Spectrogram features, the Branchformer model attained the highest accuracy scores, varying from 0.7053 to 0.7358, for frame shifts of 441, 529, and 661, respectively. This performance advantage was statistically significant, as indicated by the non-overlapping boxplot whiskers and the corresponding low p-values. Although the ResNet34 model provided commendable results within the Mel-Spectrogram feature range of 0.6795 to 0.6879, it was surpassed by the Branchformer. A trend was observed where smaller frame shifts generally led to higher accuracy across the majority of models and features, potentially due to the improved capture of continuous sound characteristics through enhanced frame-to-frame correlation. The Branchformer model maintained remarkable stability and precision across all tested frame shift sizes, which underscores its robustness in processing bowel sound data.

**Table 3:** Comparative analysis of recognition accuracy for different model architectures using different acoustic features of bowel sound signals across different frame shifts. Note that *fs* indicates the frame shift.

Model	Acoustic Feature	*ACC*_*fs*=441_	*ACC*_*fs*=529_	*ACC*_*fs*=661_
CNN	MFCC	0.6296	0.6017	0.6105
	LPC	0.6358	0.6217	0.6221
	Mel Spectrum	0.6437	0.6381	0.6295
LSTM	MFCC	0.6352	0.6586	0.6491
	LPC	0.6396	0.6359	0.6347
	Mel Spectrum	0.6642	0.6539	0.6494
CNN+LSTM	MFCC	0.6239	0.6215	0.6171
	LPC	0.6149	0.6074	0.6194
	Mel Spectrum	0.6384	0.6358	0.6281
ResNet34	MFCC	0.7014	0.6985	0.6836
	LPC	0.6749	0.6645	0.6683
	Mel Spectrum	0.6879	0.6871	0.6795
Branchformer	MFCC	0.7214	0.7158	0.7032
	LPC	0.7078	0.7012	0.6975
	Mel Spectrum	**0.7358**	**0.7224**	**0.7053**

**Fig 10:**
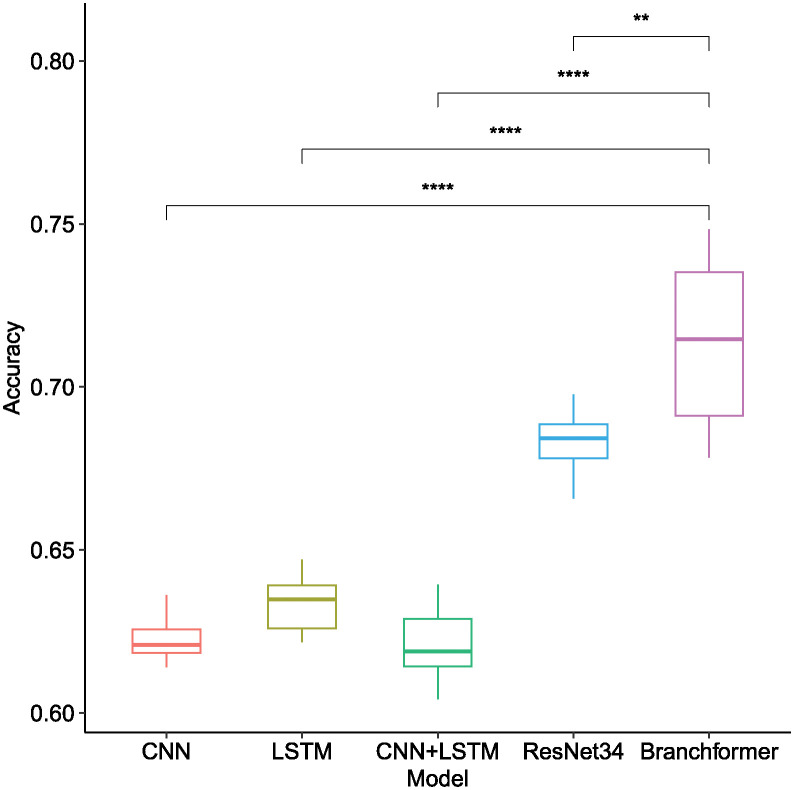
Box plot of recognition accuracy among different model architectures when utilizing different acoustic features of bowel sound signals across different feature lengths. Horizontal bars depict Mann–Whitney U tests for significance of differences in accuracy between Branchformer and other models. **p-value ≤ 0.01, ***p-value ≤ 0.001, ****p-value ≤ 0.0001.

As depicted in Table 4 and Fig 10, the Branchformer model demonstrated superior performance over alternative methods when evaluated across varying feature types and lengths. Utilizing Mel-Spectrogram features, it achieved a range of exceptional accuracy scores between 0.7352 and 0.7484 for feature lengths of 131, 164, and 199, respectively. This performance advantage was statistically significant, as illustrated by the non-overlapping boxplot whiskers and the corresponding low p-values. In comparison, the ResNet34 model achieved a lower accuracy range of 0.6781 to 0.6859 with Mel-Spectrogram features. An observed trend indicated that increased feature lengths were associated with higher accuracy for the majority of models, potentially due to the capture of more intricate sound variations. The Branchformer model displayed remarkable adaptability across all feature lengths, with a notable increase in performance for longer features, thereby highlighting its robustness in processing complex acoustic characteristics of bowel sounds.

**Table 4:** Comparative analysis of recognition accuracy for different model architectures using different acoustic features of bowel sound signals across different feature lengths. Note that *tl* indicates the feature length.

Model	Acoustic Feature	*ACC*_*tl*=131_	*ACC*_*tl*=164_	*ACC*_*tl*=199_
CNN	MFCC	0.6042	0.6185	0.6257
	LPC	0.6253	0.6278	0.6361
	Mel Spectrum	0.6140	0.6186	0.6209
LSTM	MFCC	0.6375	0.6259	0.6349
	LPC	0.6217	0.6258	0.6299
	Mel Spectrum	0.6391	0.6397	0.6470
CNN+LSTM	MFCC	0.6143	0.6194	0.6186
	LPC	0.6043	0.6048	0.6189
	Mel Spectrum	0.6288	0.6297	0.6394
ResNet34	MFCC	0.6885	0.6977	0.7054
	LPC	0.6657	0.6691	0.6842
	Mel Spectrum	0.6789	0.6781	0.6859
Branchformer	MFCC	0.6901	0.7146	0.7159
	LPC	0.6784	0.6912	0.7039
	Mel Spectrum	**0.7352**	**0.7484**	**0.7463**

**Fig 11:**
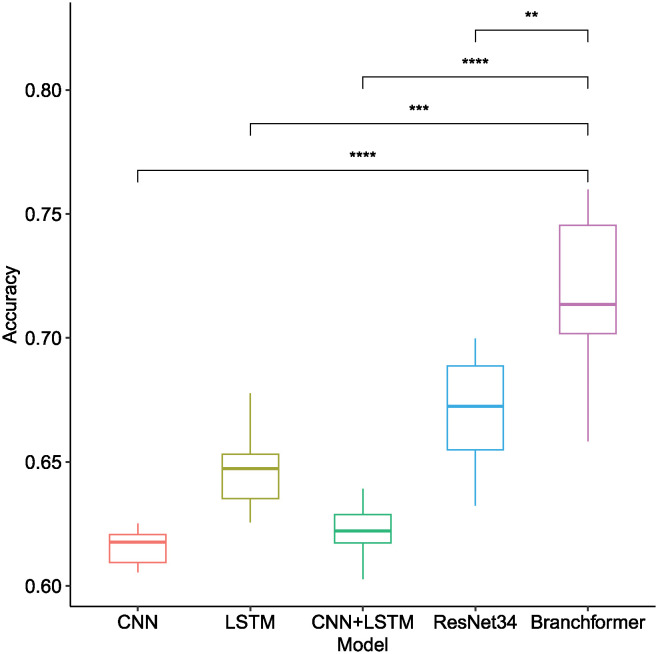
Box plot of recognition accuracy among different model architectures when utilizing different acoustic features of bowel sound signals across different window functions. Horizontal bars depict Mann–Whitney U tests for significance of differences in accuracy between Branchformer and other models. **p-value ≤ 0.01, ***p-value ≤ 0.001, ****p-value ≤ 0.0001.

As detailed in Table 5 and illustrated in Fig 11, the Branchformer model demonstrated superior performance over alternative methods when evaluated across various window functions and feature types. Utilizing Mel-Spectrogram features, the Branchformer model attained an accuracy range of 0.7135 to 0.7458 with Hanning, Hamming, and Rectangular windows, respectively. This performance advantage was statistically significant, as indicated by the non-overlapping boxplot whiskers and the corresponding low p-values. In contrast, the ResNet34 model achieved a comparatively lower accuracy range of 0.6324 to 0.6887 with Mel-Spectrogram features. The Hamming and Hanning windows typically resulted in higher accuracy compared to the Rectangular window for the majority of models, underscoring the critical role of appropriate window function selection. The Branchformer model displayed remarkable adaptability across all window functions, thereby highlighting its robustness in processing diverse acoustic features.

**Table 5:** Comparative analysis of recognition accuracy for different model architectures using different acoustic features of bowel sound signals across different window functions.

Model	Acoustic Feature	*ACC*_*Hanning*_	*ACC*_*Hamming*_	*ACC*_*Rectangle*_
CNN	MFCC	0.6233	0.6251	0.6055
	LPC	0.6198	0.6207	0.6177
	Mel Spectrum	0.6095	0.6117	0.6082
LSTM	MFCC	0.6490	0.6473	0.6257
	LPC	0.6411	0.6352	0.6286
	Mel Spectrum	0.6757	0.6776	0.6531
CNN+LSTM	MFCC	0.6221	0.6281	0.6174
	LPC	0.6287	0.6342	0.6028
	Mel Spectrum	0.6209	0.6391	0.6140
ResNet34	MFCC	0.6895	0.6997	0.6537
	LPC	0.6673	0.6724	0.6548
	Mel Spectrum	0.6887	0.6849	0.6324
Branchformer	MFCC	**0.7598**	**0.7455**	0.7017
	LPC	0.7065	0.6996	0.6583
	Mel Spectrum	0.7458	0.7411	**0.7135**

### Performance comparison of self-supervised pre-trained models

To evaluate the efficacy of self-supervised, pre-trained models in bowel sound recognition, an experimental investigation of three fine-tuning approaches was undertaken. Full parameter fine-tuning (*FP*) means full-scale adjustment of the pre-trained model; while low parameter fine-tuning is divided into two types, *LP*1 represents freezing the feature extraction layer, removing the last layer of the feature classification layer and appending a fully connected layer; *LP*2 further freezes the feature extraction layer, removes the last three layers of the feature classification layer and connects a fully connected layer.

The experimental results presented in Table 6 demonstrate that both HuBERT [39] and wav2vec 2.0 [40], which utilize transfer learning, exhibit strong performance in bowel sound recognition, particularly when employing low-parameter fine-tuning. Full parameter fine-tuning of these models yielded slightly inferior results, possibly due to the limited quantity of bowel sound data available, which may have hindered the models’ ability to fully extract relevant features. However, the implementation of low-parameter fine-tuning strategies *LP*1 and *LP*2 revealed that wav2vec 2.0 achieved marginally superior accuracy compared to HuBERT. This discrepancy may be attributed to wav2vec 2.0’s enhanced capability in capturing bowel sound features during the fine-tuning process. Notably, while HuBERT initially underperformed relative to wav2vec 2.0 in full parameter fine-tuning, it demonstrated significant improvement following low-parameter fine-tuning, suggesting greater adaptability and stability.

**Table 6:** Comparative analysis of recognition accuracy for different speech pre-trained models under different fine-tuning strategies on bowel sound data.

Model	*ACC*_*FP*_	*ACC*_*LP*1_	*ACC*_*LP*2_
HuBERT	0.7867	0.8149	0.8356
wav2vec 2.0	**0.7955**	**0.8534**	**0.8475**

Although the performance in full-parameter fine-tuning was slightly deficient, both HuBERT and wav2vec 2.0 showed significant improvements after implementing low-parameter fine-tuning, with wav2vec 2.0 slightly outperforming HuBERT. This suggests that when dealing with specialized tasks (such as bowel sound recognition) with limited data, model accuracy can be improved by adjusting the fine-tuning strategy. These experimental results also verified the feasibility and effectiveness of applying pre-trained models to the task of bowel sound recognition.

## Discussion

The pursuit of a reliable and non-invasive gastrointestinal assessment technique through auscultation has been a formidable challenge, largely due to the labor-intensive and variable nature of manual analysis. Traditional methods are not only prone to inconsistencies but also heavily dependent on the expertise and environmental conditions of clinical practitioners. The advent of bowel sound recognition technology, while promising, has encountered its own set of challenges. These include deficiencies in automatic feature extraction and the management of long-term dependencies within models such as CNNs and LSTMs. Furthermore, the scarcity and variability of high-quality bowel sound data, influenced by sensor precision, body position, and dietary habits, have added layers of complexity to the development of reliable automated systems. This study takes on these challenges by integrating advanced attention mechanisms with self-supervised pre-training strategies, aiming to bolster bowel sound recognition under data-limited conditions.

In the domain of bowel sound recognition, conventional models like CNN, LSTM, and ResNet34 encounter intrinsic challenges due to the intricate nature of bowel sound signals. These signals’ multi-frequency and non-linear attributes can surpass the feature extraction capabilities of standard CNNs and LSTMs. Moreover, the models may struggle with the long-term dependencies inherent in bowel sound signals, especially when confronted with the risk of vanishing or exploding gradients that can arise from processing extended sequences. CNNs, proficient in capturing local features, often fall short in incorporating the broader context essential for recognizing bowel sound patterns holistically. Similarly, LSTMs, despite their sequential processing prowess, may fail to fully capture the nuanced and intricate patterns present within the signals. The quality of training data is also a critical factor; insufficient or low-quality data hampers the models’ feature discernment for accurate identification. The presence of environmental noise and inter-individual physiological variation adds to the complexity, hindering the models’ generalization and precise recognition of bowel sounds. When compounded with the risks of overfitting or underfitting, these challenges highlight an urgent requirement for more advanced models capable of accommodating the complexities of bowel sound data.

Our adoption of the Branchformer architecture for bowel sound recognition represents a paradigm shift in the field. This novel deep learning model is adept at capturing both global and local features of audio signals, offering a sophisticated alternative to the conventional CNN and LSTM models that have historically been the focus of audio signal processing research. The Branchformer’s architecture is uniquely suited to the intricate temporal dynamics of bowel sounds, providing a more advanced and nuanced approach to feature extraction and pattern recognition. The consistent outperformance of the Branchformer model across a range of experimental conditions—varying sampling rates, frame lengths, shifts, feature lengths, and window functions—underscores its robustness and adaptability. This is in stark contrast to previous studies where models often faltered in capturing the subtleties and long-term patterns of bowel sound signals. The Branchformer model’s ability to maintain high accuracy across these variables heralds a potential paradigm shift in bowel sound recognition technology, suggesting the emergence of a more reliable and sensitive diagnostic tool.

Our results align with the growing body of research that underscores the importance of deep learning in medical signal processing. However, they also highlight a departure from the status quo by demonstrating the Branchformer model’s exceptional capability in bowel sound analysis. The literature [17, 23] has reported variable success with CNNs, LSTMs, and hybrid models, but none have achieved the level of accuracy we observed with the Branchformer model. This discrepancy may be attributed to the methodological advancements in our study, particularly the use of a novel model architecture that is better suited to the complex characteristics of bowel sounds.

The incorporation of self-supervised pre-training strategies in our study, as evidenced by the performance of HuBERT and wav2vec 2.0, introduces a significant advancement. These models, initially trained on vast amounts of unlabeled data, demonstrated an impressive capacity to learn generalized sound representations. The subsequent fine-tuning on bowel sound datasets, especially with low parameter fine-tuning strategies, accentuates the potential of transfer learning in enhancing model accuracy. This approach not only mitigates the challenge of limited labeled data but also underscores the adaptability of these models to specialized recognition tasks. The performance gains observed with low parameter fine-tuning highlight the strategy’s potential in scenarios where extensive labeled data may not be available. This is particularly relevant in specialized medical fields where data acquisition can be challenging. Our study’s findings suggest that with the right pre-training and fine-tuning strategies, it is possible to achieve high recognition accuracy even with limited data, thus expanding the applicability of deep learning models in such contexts.

The present study, while yielding promising results in bowel sound recognition, is not without its limitations. The experimental conditions, though meticulously designed, may not fully account for the breadth of clinical environments in which these models will ultimately be implemented. The diversity of real-world clinical settings, with varying levels of ambient noise, patient demographics, and diagnostic protocols, presents a complex backdrop against which the robustness of our models must be tested. Furthermore, the generalizability of our findings across different populations—spanning various age groups, ethnicities, and health statuses—remains an open question. It is imperative that future research endeavors diversify the dataset and broaden the scope of experimental conditions to more accurately reflect the clinical diversity and variability encountered in practice. Moreover, the current study’s dataset, while sufficient for our initial explorations, may benefit from further expansion to include a wider array of bowel sound characteristics. This expansion could involve the collection of data from a more diverse patient population, potentially revealing previously unobserved patterns and nuances in bowel sound signals. Such an enriched dataset would not only enhance the model’s predictive power but also its adaptability to different clinical scenarios.

The Branchformer model’s demonstrated efficacy in bowel sound identification opens new horizons for clinical practice, particularly in the realm of non-invasive gastrointestinal diagnostics. Its robust performance across a range of conditions suggests a strong potential for application in a variety of clinical settings, from general practice to specialized gastroenterology units. However, the successful translation of this model from the lab to the clinic will require careful consideration of its integration into existing clinical workflows and its impact on diagnostic accuracy and patient care. Future work should prioritize the expansion of the dataset to capture a more comprehensive representation of bowel sound variability. Cross-validation across diverse demographic groups will be essential to ensure the model’s broad applicability and to identify any potential biases or limitations in its predictive capabilities. Furthermore, the practical application of these models in real-world clinical settings will necessitate rigorous testing and feedback mechanisms to refine their performance and utility.

## Conclusion

This paper introduces a novel bowel sound recognition approach, which seamlessly combines the Branchformer architecture with a self-supervised pre-training strategy. Our experiments underscore the method’s considerable efficacy in bowel sound recognition, particularly in scenarios characterized by a paucity of data. The Branchformer model’s ability to concurrently process global and local features significantly bolsters its capacity for discerning bowel sound signals. The incorporation of a self-supervised pre-training strategy not only mitigates the issue of data scarcity but also tailors the model’s performance to specific tasks via fine-tuning. Furthermore, our study elucidates the influence of key parameters—such as sampling rate, frame length, frame shift, and the choice of window function—on the model’s accuracy, offering a foundation for subsequent research endeavors. While our findings are encouraging, the scope for refinement remains. Future endeavors may delve into alternative feature representations and pre-training methodologies to augment the model’s discriminative power and its applicability across diverse datasets.
